# Efficacy of Red Blood Cell Exchange as Adjunctive Treatment for Hypoxemia and Survival Rate of Patients With Severe Coronavirus-2 Disease: An Open-Labeled Phase 2 Randomized Clinical Trial

**DOI:** 10.3389/fmed.2022.899593

**Published:** 2022-07-08

**Authors:** Mohammad Aminianfar, Saeed Soleiman-Meigooni, Ramin Hamidi-Farahani, Mohammad Darvishi, Seyyed Javad Hoseini-Shokouh, Ali Asgari, Syrous Faraji-Hormozi, Maryam Asli

**Affiliations:** Infectious Diseases Research Center, AJA University of Medical Sciences, Tehran, Iran

**Keywords:** RBCs exchange, COVID-19, hypoxemia, survival rate (SR), adjunctive treatment

## Abstract

**Background:**

Severe acute respiratory syndrome (SARS) coronavirus-2 may infect red blood cells (RBCs) and impact oxygenation. We aimed to evaluate the efficacy of RBC exchange as an adjunctive treatment for hypoxemia and the survival rate of patients with severe coronavirus disease 2019 (COVID-19).

**Methods:**

In a randomized clinical trial, we divided sixty patients with severe COVID-19 into two groups. The intervention group received the standard treatment of severe COVID-19 with RBC exchange three to four times in 2 days. The control group only received the standard treatment. Our primary outcomes were improving hypoxemia in 7 days, recovery or discharge, and death in 28 days. We conducted Chi-square test, independent samples *t*-test, and Fisher’s exact test to analyze the results. The ethical committee of Aja University of Medical Sciences approved the study (IR.AJAUMS.REC.1399.054), and the Iranian clinical trial registration organization registered it (IRCT20160316027081N2).

**Results:**

Twenty-nine men and thirty-one women with a mean age of 67.5 years entered the study. The frequency of hypertension and diabetes mellitus was 86.7 and 68.3%, respectively. The most common symptoms of severe COVID-19 were dyspnea (91.6%), cough (75%), and fever (66.6%). Our results showed that hypoxemia improved in 21 of the 30 patients (70%) in the intervention group and 10 of the 30 patients (33.3%) in the control group (*P* < 0.004). The recovery and discharge rates were 19 of 30 patients (63.3%) in the intervention group and 2 of 30 patients (6.7%) in the control group (*P* < 0.001).

**Conclusion:**

The RBC exchange improved the oxygenation and survival rate in patients with severe COVID-19.

## Introduction

Efforts to find an effective treatment for severe COVID-19 have continued and mainly focused on anti-inflammatory or anticoagulation drugs. Receptors of SARS coronavirus-2 are distributed in many cells such as type 2 pneumocytes, cardiac myocytes, and intestinal epithelial cells (1). New findings also showed that the virus targets RBCs (2). Lymphopenia is the most common hematological finding in COVID-19, but hypercytokinemia and coagulopathy are two life-threatening events in severe cases of the disease (3). Several studies tried to control cytokine storms by hemofiltration or plasma replacement in severe COVID-19 (4, 5), but little data are available about RBC replacement’s efficacy in this situation. Despite normal count, deformed RBCs cannot respond to hypoxic hemoglobin levels (6). A cytokine storm leads to oxidation and fragmentation of ankyrin and spectrin in RBCs’ membrane, incomplete metabolism of lipids in saturated fatty acids, and release of glycolytic enzymes into the cytosol and glycolysis. This process led to dysfunction of RBCs despite normal count (7). Sphingosine-1-phosphate is a hypoxemic-sensitive intracellular lipid that facilitates RBC glycolysis and oxygen releases (8). A study on 10 patients with COVID-19 showed that SARS coronavirus-2 did not affect RBC oxygenation (9). Some reports showed that RBC exchange in patients with severe COVID-19 might be lifesaving, including a 22-year-old male with severe COVID-19 pneumonia and cycle cell crisis (10) and a 78-year-old male with cardiac arrest and severe hypoxemia who recovered with this method (11). In a clinical trial, we aimed to evaluate the efficacy of RBC exchange in reducing hypoxemia and increasing the survival rate of patients with severe COVID-19.

## Methods

### Study Design

This study was a randomized open-labeled phase 2 clinical trial to evaluate the efficacy of RBC exchange as an adjunctive treatment in patients with severe COVID-19 admitted to the intensive care unit at Be’sat Hospital in Tehran, Iran from 1 October 2020 to 20 January 2021.

### Participants and Intervention

Our participants were patients over 18 years old with severe COVID-19. The inclusion criteria were positive polymerase chain reaction for SARS coronavirus-2 infection, presence of at least three severity disease indexes including baseline respiratory rate above thirty per minute or oxygen saturation level less than 80% on room air, decreased level of consciousness, pulmonary ground glass opacity of over 50% in computed tomography, and presence of at least one underlying disease such as diabetes, hypertension, ischemic heart disease, chronic lung disease, or obesity with a body mass index of more than 25 kg/m^2^. The exclusion criteria included myocardial infarction, pulmonary thromboembolism, intubation, severe transfusion-related adverse events, baseline hemoglobin level of less than 7 mg/dl, and incomplete treatment due to ceased consent. All of the patients entered the study before intubation and mechanical ventilation.

### Study Protocol

After assessing eligibility and randomizing with the permuted block randomization method, we explained the study protocol to the patients or their first-degree relatives to participate by their decision. In the intervention group, we prescribed the standard treatment protocol for severe COVID-19 based on the “Eighth Edition of COVID-19 Diagnosis and Treatment” announced by the Iranian Ministry of Health (12), including interferon β-1b or β-1a, dexamethasone, and prophylactic anticoagulants, concurrent with RBC exchange. In the control group, we only prescribed the standard treatment protocol. For RBC exchange, we phlebotomized 400–500 cc of whole blood equal to 200–250 cc of peripheral RBC. Then we transfused the same volume of packed RBCs after isogroup and crossmatch testing and crystalloid liquids such as sodium chloride 0.9%. We repeated this process three to four episodes in 2 days as needed (Table 1).

**TABLE 1:** Technique of red blood cell (RBC) exchange in the intervention group.

Baseline hemoglobin level	Phlebotomy volume	Patient’s weight	Phlebotomy repeat number	Total RBC exchange volume	Subgroup
≥14 mg/dl in men ≥12 mg/dl in women	500 cc	≥70 kg	Four	1,000 CC	1
		<70 kg	Three	750 CC	2
12–14 mg/dl in men 10–12 mg/dl in women	450 cc	≥70 kg	Four	900 CC	3
		<70 kg	Three	675 CC	4
<12 mg/dl in men <10 mg/dl in women	400 cc	≥70 kg	Four	800 CC	5
		<70 kg	Three	600 CC	6
					

We assessed vital signs, transfusion-related adverse events, and O_2_-saturation daily and monitored laboratory changes every other day. We finally analyzed the data with SPSS software version 26 (IBM Corporation) by chi-square test, independent samples *t*-test, and Fischer exact test at a significant level of 95%.

### Outcome Measures

The primary outcomes in our study were permanent increase in oxygen saturation of more than 10% from the baseline in 7 days, recovery and discharge of the patients, and death during the first 28 days after admission.

### Ethical Issues

The ethical committee of Aja University of Medical Sciences, Tehran, Iran, approved this study with the approval code IR.AJAUMS.REC.1399.054 on 6 June 2020. We registered this study in the Iranian clinical trials organization with the registration code IRCT20160316027081N2.

## Results

**FIGURE 1:**
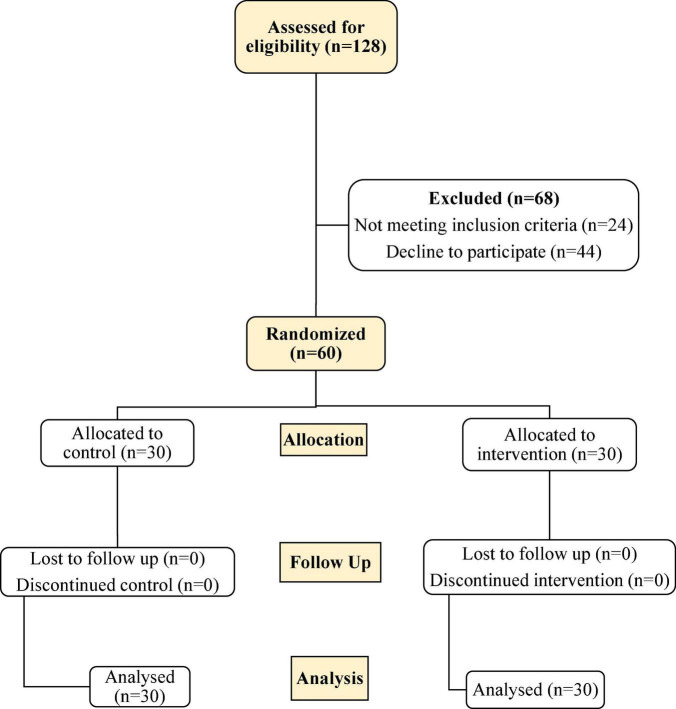
Flow diagram of the study.

**FIGURE 2:**
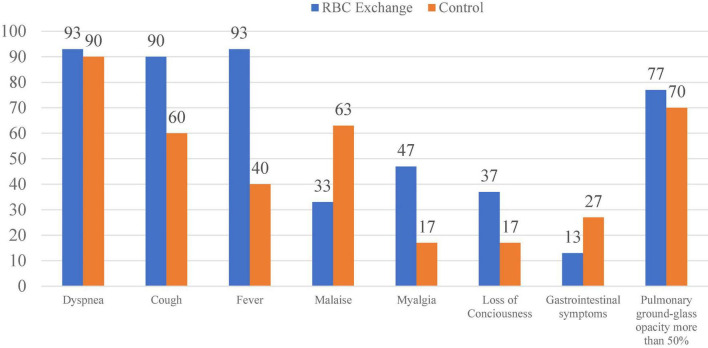
Percentage of symptoms at the time of admission in the two groups.

We divided the 60 patients into two groups consisting of thirty patients in each arm by the permuted block randomization method (Figure 1). Among the patients, 29 were men, and 31 were women. The mean age of the patients was 67.3 + 12 years (24–90 years), and the mean body mass index (BMI) was 25.5 ± 3 (20–31), consisting of 25.8 ± 2.9 in the male and 25.2 ± 3.1 in the female patients (*P* = 0.47). The mean hemoglobin level was 12.9 ± 1.6 g/dl in the men and 11.7 ± 1.5 g/dl in the women (*P* = 0.008). The frequency of preexisting diseases was 52 of 60 (86.7%) for hypertension, 41 of 60 (68.3%) for diabetes mellitus, 25 of 60 (45%) for ischemic heart disease, and 11 of 60 (18.3%) for chronic pulmonary disease. Figure 2 compares the patients’ symptoms on admission, and Table 2 compares the demographic characteristics, preexisting diseases, clinical symptoms, laboratory findings, and primary outcomes between the two groups. The most common symptoms of the patients were shortness of breath (91.6%), cough (75%), and fever (66.6%). We did not find any transfusion-related adverse event during the study. On the 7th day of admission, the mean hemoglobin level was not different between the two groups (11.2 ± 1.7 mg/dl in the intervention group and 10.7 ± 1.8 g/dl in the control group, *P* = 0.253).

**TABLE 2:** Main data between the two groups (* statistically significant at *P* < 0.05).

Group	RBC exchange (*n* = 30)	Control (*n* = 30)	*P*-value
		
Variable	Mean ± SD	Mean ± SD	
Age (years)	64.5 ± 13.2	70.2 ± 10.2	0.067
Male/Female ratio	18/12	11/19	0.120
Body mass index (BMI)	26.2 ± 2.7	24.8 ± 3.2	0.071
Systolic blood pressure on 1th Day	130.7 ± 14.5	132.2 ± 15.2	0.710
Systolic blood pressure on 7th day	119.7 ± 18.4	119.6 ± 21.6	0.989
Pulse rate on the 1st day	83.6 ± 14.5	91.1 ± 16.5	0.067
Pulse rate on the 7th day	103.4 ± 16.2	108.9 ± 13.8	0.200
SpO_2_ on the 1st day	85.1 ± 3.8	84.7 ± 5.7	0.752
SpO_2_ on the 3rd day	87 ± 3.4	81.3 ± 6.8	<0.001*
SpO_2_ on the 7th day	88.4 ± 5.9	75.4 ± 8.1	<0.001*
Acidity (PH) on the 1st day	7.37 ± 0.05	7.36 ± 0.07	0.436
Acidity (PH) on the 7th day	7.40 ± 0.10	7.26 ± 0.1	<0.001*
LDH on the 1st day	627 ± 242	532 ± 210	0.120
Primary WBC (per ml)	7,936 ± 3,718	6,943 ± 3,866	0.315
Primary hemoglobin (per mg/dl)	12.7 ± 1.6	11.9 ± 1.7	0.063
Primary platelets (per ml)	186,000 ± 58,000	181,000 ± 63,000	0.792
Primary CRP	50.6 ± 18.3	51.2 ± 11.2	0.886
Hypertension	26 (86.7%)	26 (86.7%)	1.000
Diabetes mellitus	21 (70%)	20 (66.7%)	1.000
Coronary artery disease	11 (36.7%)	16 (53.3%)	0.299
Chronic pulmonary diseases	7 (23.3%)	4 (13.3%)	0.506

**FIGURE 3:**
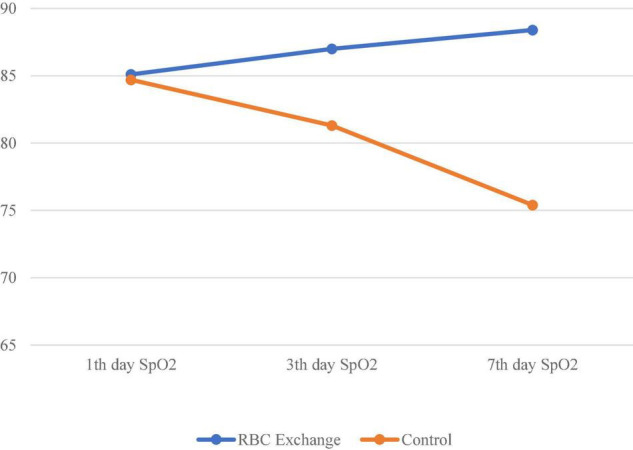
Comparison of the mean trend of oxygen saturation on the 1st, 3rd, and 7th days of admission in the two groups.

As shown in Table 1, the mean SpO_2_ on the 3rd and 7th days of admission was significantly improved in the intervention group by *t*-test (*P* < 0.001, Figure 3). Furthermore, the survival rate on the 7th day of admission was 19 of the 30 patients (63.3%) in the intervention group compared to the 2 of the 30 patients (6.7%) in the control group, and was statistically different by Fischer exact test (*P* < 0.001, risk ratio = 24.18). All the survivors were still alive after 28 days.

## Discussion

Our study was one of the first to evaluate the effect of RBC exchange on the survival rate of patients with COVID-19. As in most studies, our subjects were old, with a mean age of 67 years, and most had preexisting conditions. Severe COVID-19 occurred in 13–24% of all cases with SARS coronavirus-2 infection. The mortality rate of COVID-19 is estimated at 1% in the general population and more than 40% in severe diseases. Many patients with severe COVID-19 still die despite receiving complex treatments, including antivirals, anticoagulants, and corticosteroids (13, 14). Therefore, we still need new complementary treatments to reduce mortality.

Recently, more attention has been paid to RBC’s function in COVID-19. SARS coronavirus-2 attaches to different blood cells such as WBCs and RBCs and stimulates coagulation and inflammatory cascades (15). One study showed that the serum of patients with COVID-19 could agglutinate RBCs (16). Another study on 20 patients with COVID-19 found that the most common RBC changes were polychromasia, basophilic stippling, autoagglutination, and schistocytes, which are compatible with microangiopathy (17). Also, COVID-19 reduced RBCs’ membrane elasticity and deformed them, which predisposes them to microthrombosis (18). SARS coronavirus-2 directly affects peripheral RBCs or bone marrow erythroblasts or indirectly destroys RBCs because of hemolytic anemia and coagulopathy. These lead to increase in number of old RBCs in circulation and delays in clearance. A study on 280 recovered and 86 deceased patients with COVID-19 showed that red cell distribution width was significantly higher in deceased patients (81.4% vs. 41.8%, *P* < 0.001) (19). Scientists also hypothesized that SARS coronavirus-2 destroyed the RBC membrane, decreased vasodilation and oxygen delivery in tissues, and contributed to persistent symptoms such as fatigue in recovered patients (20). Early administration of erythropoietin or blood transfusion is recommended to treat hypoxemia in patients with COVID-19 (21). Also, initial treatment with dexamethasone and euthyroid cells results in downregulation of ACE-2 and TMPRSS2 in RBCs and decreases infection with the virus (22).

The mean time for the onset of symptoms to admission in our patients was 6 days, and we expected many of our patients to pass the viral phase of the disease and develop a cytokine storm. We phlebotomized whole blood and substituted it with packed RBCs and crystalloid liquids. We think that this procedure replaced infected RBCs with fresh ones and removed hyperimmune plasma containing pathogenic cytokines. However, we did not explicitly examine old RBCs and did not measure specific cytokines before and after the intervention. Numerous studies have shown that plasmapheresis leads to recovery in patients with severe COVID-19 and cytokine storms (23, 24). However, there is still no FDA-approved guideline in this case. In our study, RBC exchange improved oxygenation and reduced the mortality rate in patients with severe COVID-19 pneumonia. Historically, replacement an infected or non-functional organ has been widely performed in some advanced diseases such as liver cirrhosis, heart failure, and chronic renal failure. RBC exchange is also a type of organ transplant widely conducted in kernicterus (25), severe falciparum malaria in the form of black water fever (26), sepsis and septic shock (27), sickle cell crisis (28–30), and methemoglobinemia (31). We suggest that RBC exchange can be conducted as adjunctive treatment in patients with severe COVID-19 pneumonia.

## Conclusion

RBC exchange is a new therapeutic approach as adjunctive treatment in some patients with severe COVID-19. This treatment helps oxygenation by physical removal of non-functional RBCs and substitutes them with new RBCs. We suggest for other scientists to try this treatment for better evaluation and consistency.

### Limitations

Our limitations consisted of lack of hematologic laboratory examination of phlebotomized RBCs, inequality of male to female ratio in the two groups, unavailability of some information such as inflammatory biomarkers like IL-6 and ferritin, cardiac ejection fraction ratio, and pulmonary CT-angiogram in all the patients.

## Data Availability Statement

The raw data supporting the conclusions of this article will be made available by the authors, without undue reservation.

## Ethics Statement

The studies involving human participants were reviewed and approved by the Aja University of Medical Sciences. The patients/participants provided their written informed consent to participate in this study.

## Author Contributions

MA designed the study protocol, reviewed the literature, and wrote the primary draft of the manuscript. SS-M contributed to the treatment of the patients, reviewed the literature, and wrote the primary and final manuscript. RH-F, MD, SH-S, AA, SF-H, and MA contributed to the treatment of the patients, reviewing the literature, and wrote the primary manuscript. All authors contributed to the article and approved the submitted version.

## Conflict of Interest

The authors declare that the research was conducted in the absence of any commercial or financial relationships that could be construed as a potential conflict of interest.

## Publisher’s Note

All claims expressed in this article are solely those of the authors and do not necessarily represent those of their affiliated organizations, or those of the publisher, the editors and the reviewers. Any product that may be evaluated in this article, or claim that may be made by its manufacturer, is not guaranteed or endorsed by the publisher.

## References

[1] Li MY Li L Zhang Y Wang XS. Expression of the SARS-CoV-2 cell receptor gene *ACE2* in a wide variety of human tissues. *Infect Dis Poverty.* (2020) 9:45. 10.1186/s40249-020-00662-x 32345362 PMC7186534

[2] Cosic I Cosic D Loncarevic I. RRM prediction of erythrocyte Band3 protein as alternative receptor for SARS-CoV-2 virus. *Appl Sci.* (2020) 10:4053.

[3] Letícia de Oliveira Toledo S Sousa Nogueira L das Graças Carvalho M Romana Alves Rios D de Barros Pinheiro M. COVID-19: review and hematologic impact. *Clin Chim Acta.* (2020) 510:170–6. 10.1016/j.cca.2020.07.016 32659224 PMC7351669

[4] Asgharpour M Mehdinezhad H Bayani M Zavareh MSH Hamidi SH Akbari R Effectiveness of extracorporeal blood purification (hemoadsorption) in patients with severe coronavirus disease 2019 (COVID-19). *BMC Nephrol.* (2020) 21:356. 10.1186/s12882-020-02020-3 32819292 PMC7439633

[5] Ronco C Bagshaw SM Bellomo R Clark WR Husain-Syed F Kellum J Extracorporeal blood purification and organ support in the critically ill patient during COVID-19 pandemic: expert review and recommendation. *Blood Purif.* (2021) 50:17–27. 10.1159/000508125 32454500 PMC7270067

[6] Kubánková M Hohberger B Hoffmanns J Fürst J Herrmann M Guck J Physical phenotype of blood cells is altered in COVID-19. *Biophys J.* (2021) 120:2838–47. 34087216 10.1016/j.bpj.2021.05.025 PMC8169220

[7] Thomas T Stefanoni D Dzieciatkowska M Issaian A Nemkov T Hill RC Evidence for structural protein damage and membrane lipid remodelling in red blood cells from COVID-19 patients. *J Proteome Res.* (2020) 19:4455–69. 33103907 10.1021/acs.jproteome.0c00606 PMC7640979

[8] Sun K Zhang Y D’Alessandro A Nemkov T Song A Wu H Sphingosine-1-phosphate promotes erythrocyte glycolysis and oxygen release for adaptation to high-altitude hypoxia. *Nat Commun.* (2016) 7:12086. 10.1038/ncomms12086 27417539 PMC4947158

[9] Park KC Donovan K McKechnie S Ramamurthy N Klenerman P Swietach P. Single-cell oxygen saturation imaging shows that gas exchange by red blood cells is not impaired in COVID-19 patients. *Br J Haematol.* (2020) 190:e229–32. 10.1111/bjh.17025 32678950 PMC7405117

[10] Okar L Aldeeb M Yassin MA. The role of red blood cell exchange in sickle cell disease in patients with COVID-19 infection and pulmonary infiltrates. *Clin Case Rep.* (2020) 9:337–44. 10.1002/ccr3.3526 33362923 PMC7753272

[11] Ejigu T Patel N Sharma A Vanjarapu JMR Nookala V. Packed red blood cell transfusion as a potential treatment option in COVID-19 patients with hypoxemic respiratory failure: a case report. *Cureus.* (2020) 12:e8398. 10.7759/cureus.8398 32637278 PMC7331927

[12] Abtahi T Ashrafi F Iraj B Bidari A Biglari A. *Eighth version of Diagnosis and Treatment Protocol of Covid-19, by Ministry of Health and Medical Education*. Available online at: https://irimc.org/news/id/45316

[13] Hu Y Sun J Dai Z Deng H Li X Huang Q Prevalence and severity of corona virus disease 2019 (COVID-19): a systematic review and meta-analysis. *J Clin Virol.* (2020) 127:104371. 10.1016/j.jcv.2020.104371 32315817 PMC7195434

[14] Alimohamadi Y Tola HH Abbasi-Ghahramanloo A Janani M Sepandi M. Case fatality rate of COVID-19: a systematic review and meta-analysis. *J Prev Med Hyg.* (2021) 62:E311–20. 10.15167/2421-4248/jpmh2021.62.2.1627 34604571 PMC8451339

[15] Hacking SM. Red blood cell exchange for SARS-CoV-2: a gemini of therapeutic opportunities. *Med Hypotheses.* (2020) 144:110227. 10.1016/j.mehy.2020.110227 33254534 PMC7467009

[16] Kruse RL Huang Y Smetana H Gehrie EA Amukele TK Tobian AAR A rapid, point-of-care red blood cell agglutination assay detecting antibodies against SARS-CoV-2. *Biochem Biophys Res Commun.* (2021) 553:165–71. 10.1016/j.bbrc.2021.03.016 33773139 PMC7959259

[17] Berzuini A Bianco C Migliorini AC Maggioni M Valenti L Prati D. Red blood cell morphology in patients with COVID-19-related anaemia. *Blood Transfus.* (2021) 19:34–6. 10.2450/2020.0242-20 32955421 PMC7850925

[18] Renoux C Fort R Nader E Boisson C Joly P Stauffer E Impact of COVID-19 on red blood cell rheology. *Br J Haematol.* (2021) 192:e108–11. 10.1111/bjh.17306 33410504

[19] Soni M Gopalakrishnan R. Significance of RDW in predicting mortality in COVID-19-An analysis of 622 cases. *Int J Lab Hematol.* (2021) 43:O221–3. 10.1111/ijlh.13526 33774907 PMC8250958

[20] Misiti F. SARS-CoV-2 infection and red blood cells: implications for long term symptoms during exercise. *Sports Med Health Sci.* (2021) 3:181–2. 10.1016/j.smhs.2021.07.002 34337552 PMC8302835

[21] Zubieta-Calleja G Zubieta-DeUrioste N. Pneumolysis and “silent hypoxemia” in COVID-19. *Ind J Clin Biochem.* (2021) 36:112–6. 10.1007/s12291-020-00935-0 33191989 PMC7652053

[22] Shahbaz S Xu L Osman M Sligl W Shields J Joyce M Erythroid precursors and progenitors suppress adaptive immunity and get invaded by SARS-CoV-2. *Stem Cell Rep.* (2021) 16:1165–81. 10.1016/j.stemcr.2021.04.001 33979601 PMC8111797

[23] Krzych ŁJ Putowski Z Czok M Hofman M. What is the role of therapeutic plasma exchange as an adjunctive treatment in severe COVID-19: a systematic review. *Viruses.* (2021) 13:1484. 10.3390/v13081484 34452349 PMC8402853

[24] Wardhani SO Fajar JK Soegiarto G Wulandari L Maliga HA Ilmawan M The association between therapeutic plasma exchange and the risk of mortality among patients critically ill with COVID-19: a meta-analysis. *F1000Research.* (2021) 10:1280. 10.12688/f1000research.74972.1 35083038 PMC8749910

[25] Ballot DE Rugamba G. Exchange transfusion for neonatal hyperbilirubinemia in Johannesburg, South Africa, from 2006 to 2011. *Int Sch Res Notices.* (2016) 2016:1268149. 10.1155/2016/1268149 27382636 PMC4897111

[26] Auer-Hackenberg L Staudinger T Bojic A Locker G Leitner GC Graninger W Automated red blood cell exchange as an adjunctive treatment for severe Plasmodium falciparum malaria at the Vienna General Hospital in Austria: a retrospective cohort study. *Malaria J.* (2012) 11:1–7. 10.1186/1475-2875-11-158 22564543 PMC3447647

[27] Arango-Granados MC Umaña M Sánchez AI García AF Granados M Ospina-Tascón GA. Impact of red blood cell transfusion on oxygen transport and metabolism in patients with sepsis and septic shock: a systematic review and meta-analysis. *Rev Bras Ter Intensiva.* (2021) 33:154–66. 10.5935/0103-507X.20210017 33886865 PMC8075342

[28] Asma S Kozanoglu I Tarım E Sarıturk C Gereklioglu C Akdeniz A Prophylactic red blood cell exchange may be beneficial in the management of sickle cell disease in pregnancy. *Transfusion.* (2015) 55:36–44. 10.1111/trf.12780 25070465

[29] Chou ST Alsawas M Fasano RM Field JJ Hendrickson JE Howard J American Society of Hematology 2020 guidelines for sickle cell disease: transfusion support. *Blood Adv.* (2020) 4:327–55. 10.1182/bloodadvances.2019001143 31985807 PMC6988392

[30] Nguyen V Alcius P Peles S Hodgin K. A fresh breath of oxygen: red blood cell exchange transfusion in sickle cell and COVID-19. *Clin Case Rep.* (2021) 9:e04655. 10.1002/ccr3.4655 34466242 PMC8385183

[31] Khetarpal A Kotwal U. Role of Automated therapeutic red cell exchange in thesetting of acute methemoglobinemia: our experience. *Ind J Hematol Blood Transfus.* (2018) 34:143–5. 10.1007/s12288-017-0832-x 29398814 PMC5786626

